# Optimization Extraction of Shikonin Using Ultrasound-Assisted Response Surface Methodology and Antibacterial Studies

**DOI:** 10.1155/2020/1208617

**Published:** 2020-07-29

**Authors:** Xiang-Yue Huang, Hua-Lin Fu, Hua-Qiao Tang, Zhong-Qiong Yin, Wei Zhang, Gang Shu, Li-Zi Yin, Ling Zhao, Xiao-Rong Yan, Ju-Chun Lin

**Affiliations:** ^1^Department of Pharmacy, College of Veterinary Medicine, Sichuan Agricultural University, Chengdu, China; ^2^The People's Hospital of Ya'an, Ya'an, China

## Abstract

The objectives of this study were to develop and optimize ultrasound-assisted extraction (UAE) for shikonin from *Arnebia euchroma* using response surface methodology (RSM) and to evaluate the antimicrobial activity of shikonin. The maximum yield of shikonin was 1.26% under the optimal extraction conditions (ultrasound power, 93 W; time, 87 min; temperature, 39°C; and liquid-solid ratio, 11 : 1). Shikonin showed inhibitory activity against standard strains and clinical isolates to varying extents (MICs ranging from 128 to 1024 *μ*g/mL, MBCs ranging from 256 to 2048 *μ*g/mL), and it was more effective for Gram-positive bacteria as indicated by lower MIC and MBC values. Time-kill curves revealed that antibacterial activity of shikonin exhibited a dose-response relationship. In summary, via this study, we identified ultrasound-assisted RSM as the optimal extraction method for shikonin, which is a potential material for the treatment of bacterial infections.

## 1. Introduction

**Figure 1:**
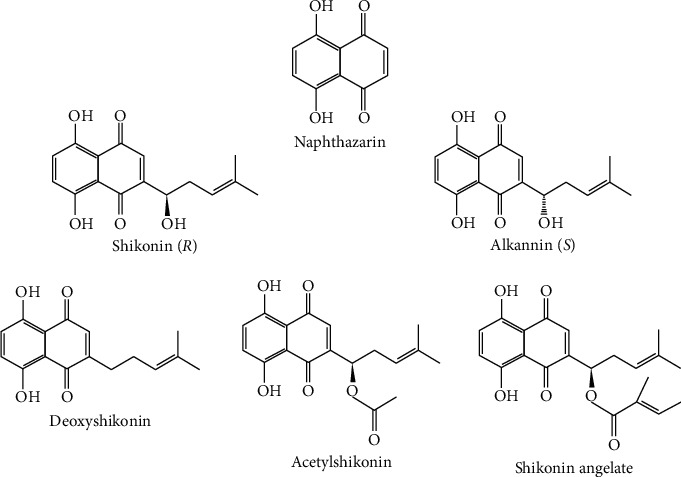
Chemical structures of shikonin derivatives.

*Arnebia euchroma* is a traditional Asian medicine for the treatment of ulcers, burns, chicken pox, and hepatitis [1]. The medicinal component of *A. euchroma* is *Radix Arnebiae.* Numerous natural comfrey compounds can be isolated from different comfrey species by different methods, such as shikonin, deoxyshikonin, diacetylshikonin, 1-methoxy-acetylshikonin, *β*,*β*′-dimethylacrylshikonin, 2,3-teracrylshikonin, *β*-hydroxyisovalerylshikonin, *β*-HIVS, *β*,*β*-dimethylacrylalkannin, acetylalkannin, *β*-acetoxy-isovalerylalkannin, and *β*-hydroxy-isovalerylalkannin, which are the major active constituents of naphthoquinones (Figure 1).

Pharmacological studies have indicated that shikonin exerts several beneficial effects such as anti-inflammatory, antimicrobial, antiallergic, and wound-healing activities [2]. In addition, shikonin is an ester-soluble pigment that is soluble in alcohol, organic solvents, and vegetable oils; therefore, an organic solvent was selected for the extraction of shikonin. In previous studies, immersion extraction, ethanol percolation, microwave-assisted extraction, and heat reflux extraction [3–5] were the common extraction methods for shikonin; however, these methods have some drawbacks such as severe environmental hazards, increased solvent consumption, long extraction time, and possible degradation of the target compounds. Many studies indicated that shikonin is thermally unstable and is readily degraded during various extraction processes when the temperature exceeds 60°C [6,7]. Ultrasound-assisted extraction (UAE) is a useful technology for highly efficient and rapid extraction of compounds [8,9]. At present, UAE provides a broad range of benefits such as reduced extraction time, lower consumption of organic solvents, and increased extraction yield of target components; moreover, the conditions of UAE, including temperature, ratio of material to liquid, and extraction time, significantly influence the extraction efficiency of traditional Chinese medicinal compounds. Although the extraction time is shortened when ultrasonic extraction is used, compared with the traditional extraction method, the active components are easy to be inactivated, so the extraction time should not be too long. Response surface methodology (RSM) is a group of statistical and mathematical techniques applied widely to improve and optimize experiments that can determine the optimum conditions and reduce the number of experimental runs required [10–13]. The aim of the present study was to develop a UAE method for the extraction of shikonin from *A. euchroma* and to obtain suitable extraction conditions for production, energy saving, and convenience. In addition, we aimed to evaluate the antibacterial activity of shikonin *in vitro*.

## 2. Materials and Methods

### 2.1. Plant Material and Chemical Reagents

*A. euchroma* roots were purchased from Hehuachi Medicinal Materials Market, (Chengdu, China). Shikonin standard (HPLC grade, purity ≥98%) was purchased from the National Institute for the Control of Pharmaceutical and Biological Products (Beijing, China). Anhydrous ethanol (AR), methanol (AR, HPLC), petroleum ether (boiling point 60–90°C AR), acetic acid, sodium hydroxide, sodium chloride, lecithin, and cholesterol were purchased from Beijing Chemical Reagents Co. (Beijing, China). A high-performance liquid chromatograph (Agilent 1200), ultralow-temperature freezer (Revco ULT-1786-6V), Ultrasonic and microwave coextraction apparatus (CW-2000), and laboratory incubator (SMI12-2) were used.

### 2.2. Extraction of Shikonin

#### 2.2.1. Ultrasound Extraction and Single-Factor Experiment

Radix *A. euchroma* (10 g) was placed in a beaker and filled with a specified volume of ethanol solution (95%). The single-factor experimental conditions were established as follows: liquid-solid ratio (A), 1 : 1 to 20 : 1; ultrasonic time (B), 45–105 min; extraction temperature (°C), 10–50°C, and ultrasonic power (D), 30–150 W. The extract was concentrated by evaporation under vacuum (50°C) in a rotary evaporator and was freeze-dried. The effects of each factor were investigated by analyzing shikonin yield.

#### 2.2.2. Detection Conditions of Shikonin by HPLC

The concentration of shikonin was measured by HPLC. The HPLC conditions were as follows: chromatographic column-Kromasil C18 (250 mm × 4.6 mm, 5 *μ*m), the mobile phase consisted of a mixture of methanol-water (90 : 10), a flow rate of 1 mL/min, a detection wavelength of 516 nm, a column temperature of 25°C, and an injection volume of 20 *μ*L [14].

#### 2.2.3. Parameter Optimization by Response Surface Methodology

In this study, RSM was used to identify the effects of optimum parameters of four variables, liquid-solid ratio (A), ultrasonic time (B), extraction temperature (C), and ultrasonic power (D), on the yield of shikonin. To estimate the model coefficients, a three-factor-three-level Box-Benhnken experiment was performed (29 runs), and the order of the experiments was randomized (Table 1).

For the statistical calculations, the variables were coded in accordance with the following equation:

(1) $$\begin{array}{c} x_{i}=\frac{X_{i}-X_{0}}{\Delta X},\;\left(i=1,2,3\right), \end{array}$$

where *x*_i_ represents the coded value, *X*_*i*_ is the corresponding actual value, *X*_0_ is the actual value in the center of the domain, and ∆*X* is the step-change value. From the experimental data, a second-order polynomial equation was used to express the responses as a function of the independent variables as follows:

(2) Y=β0+∑i=14βXii+∑i=14βiiXi2+∑i=13∑j=i+14βijXiXj,

where *Y* represents the dependent variable (shikonin yield), *β*_0_ is the constant coefficient, *β*_*i*_, *β*_*ii*_, and *β*_*ij*_ represent the model coefficients of the linear, quadratic, and interaction effects of the variables, respectively; *X*_*i*_ and *X*_*j*_ are the coded independent variables. The analysis of the experimental design data and the calculation of the predicted responses were computed by using SPSS Statistics 22.0. Additional confirmatory experiments were subsequently conducted to verify the reliability of the statistical experimental design.

### 2.3. Conversion and Refinement of Shikonin

The crude extract (0.2 g) was weighed, 100 mL of petroleum ether was added to dissolve the crude extract, and 100 mL of 2% NaOH solution was added to the mixture. The mixture was heated in a water bath at 40°C and allowed to stand for at least 4 h, and the solution was transferred to the separatory funnel. The underlying alkaline water extract was removed and filtered; and acetic acid was added to the solution to reach a pH between 5 and 6, and the solution was allowed to stand for 12 h. Finally, the solution was extracted by petroleum ether until the solution was colorless; then, the petroleum ether mixture was removed by rotary evaporation and dried at 50°C to obtain shikonin. The shikonin yield was measured by HPLC, and the purity of shikonin was calculated [15].

### 2.4. Antibacterial Activity of Shikonin In Vitro

#### 2.4.1. Bacterial Strains and Cultivation

*Escherichia coli* (ATCC 25922), *Salmonella pullorum* (C79-13), *Pseudomonas aeruginosa* (ATCC 9027), *Staphylococcus aureus* (ATCC 25923), and *Streptococcus* agalactiae (ATCC 13813) were purchased from BeNa Culture Collection (Peking, China). Clinical isolates of *E. coli* isolates (EYAC15-12, 15-30, 15-64), *S. aureus* isolates (staYAC16-5, 16-15, 16-23), *Salmonella* isolates (SYAC16-10, 16-14, 16-41) were isolated from sick chicken and provided by the National animal experimental teaching demonstration center, Sichuan Agricultural University, Chengdu, China. These strains were grown in Luria-Bertani (LB) medium overnight at 37°C. A bacterial suspension of 1 × 10^6^ CFU/mL in LB was used in the antibacterial experiment in vitro.

#### 2.4.2. Measurement of the MIC and MBC of Shikonin

For the MIC determination, the broth dilution method was chosen [16]. Shikonin was diluted by twofold serial dilutions from 2048 *μ*g/mL to 2 *μ*g/mL after the addition of bacterial suspension (approximately 1–2 × 10^5^ CFU/mL). Simultaneously, the microbial and TSB broth control were established. The final volume for each tube was 1 mL in glass tubes. After incubation (37°C, 16–24 h), the lowest concentration without visible bacterial turbidity was the MIC. To determine the MBC, 100 *μ*L of the culture was transferred and inoculated on Muller–Hinton agar (MHA, 37°C, 24 h), as previously described [17]. The minimum concentration without bacterial growth was considered the MBC.

#### 2.4.3. Analysis of the Time-Kill Curves

Time-kill curves were constructed by a previously published method [18]. Briefly, freshly prepared colonies were resuspended in 10 mL Mueller–Hinton Broth (MH) and incubated in a shaking water bath (37°C, 180 rpm) for 1–2 h. The cultures were then diluted to approximately 5 × 10^6^ CFU/mL. Shikonin was added to the prepared bacterial suspensions so that the final shikonin concentrations were 1/2×, 1×, or 2× MIC of shikonin. A growth control, without shikonin, was also included. Conical flasks were incubated in a shaking water bath (37°C, 180 rpm), and viability counts were performed after incubation for 3, 6, 9, 12, and 24 h by removing 1 mL of the culture, diluted as appropriate, and plating 100 *μ*L on MHA. MHA plates were incubated at 37°C for a minimum of 18 h. The colonies were counted, and the results were recorded as the number of CFU/mL. The experimental results are expressed as log_10_ CFU/mL of time as a time-kill curve [19].

### 2.5. Statistical Analysis

Statistical analyses were computed by using SPSS Statistics 22.0 software. All data were analyzed by using analysis of variance (ANOVA followed by Tukey's test) for multiple comparisons and are expressed as the mean ± SD. A *p* value of less than 0.05 was considered statistically significant.

## 3. Results and Discussion

### 3.1. Single-Factor Test

**Figure 2:**
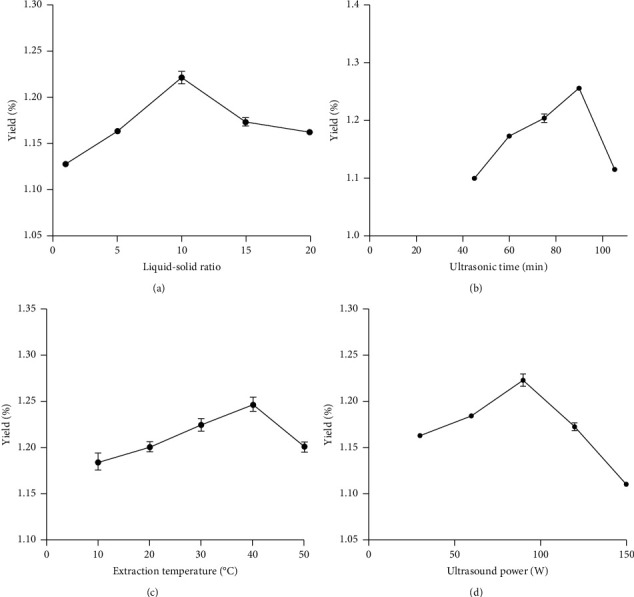
The yields of shikonin in single-factor experiments. Liquid-solid ratio (a), ultrasonic time (b), extraction temperature (c), and ultrasonic power (d).

Experiments were designed to determine the effect of each factor on the total pigment yield of *A. euchroma.* As shown in Figure 2, the yield increased with an increase in the liquid-solid ratio, and the highest yield was obtained when the liquid-solid ratio was 11 : 1. Hence, a liquid-to-solid ratio range of 5 : 1–15 : 1 was used for the subsequent experiment. The yield of shikonin markedly increased when the extraction time was 45–90 min, and the yield of shikonin tended to become more stable; to save time, a 75–105 min treatment time was selected for further optimization experiments. The production of shikonin increased with increasing extraction temperature and ultrasonic power, and it did not decrease until it reached the values of 40°C and 90 W. Therefore, the appropriate range of the extraction temperature and ultrasound power was 30–50°C and 60–120 W, respectively.

### 3.2. Statistical Analysis and Model Fitting Using RSM

Similar to a previous study [20], 29 experiments were designed and carried out using Box-Benhnken experiment for RSM, and this was applied to optimize ultrasonic conditions. The results are listed in Table 2. As shown by the regression analysis of the experimental data, the extraction efficiency can be explained by the following equation:

(3) $$\begin{array}{c} \text{yield}=1.25+0.030A-0.014B-0.0014C+0.006\;D-0.0093AB-0.0095AC\\ +0.0045A\;D+0.003BC-0.00175B\;D+0.012C\;D-0.067A^{2}-0.042B^{2}-0.012C^{2}-0.031D^{2}. \end{array}$$

*A*, *B*, *C*, and *D* are the coded variables for the liquid-solid ratio, ultrasonic time (min), extraction temperature (°C), and ultrasonic power (W), respectively. As shown in Table 3, the *F*-value of the model was 51.64, and the *p* value was <0.0001, which suggested that the model was significant. The coefficients of the models for these four components were significant for *p* values less than 0.05, but the other coefficients were not significant (*p* > 0.05). For shikonin yield, the factors were ranked by significance in the following order: *A* > *B* > *D* > *C*. In addition, the *p* value for the lack of fit was 0.1512 (*p* > 0.05), indicating that the regression equation was more in line with the actual operation. These results indicated that the regression equation could be used instead of the real point of the experiment results; thus, it could be used to analyze and predict the yield of shikonin. The value of the determination coefficient (*R*^2^ = 0.9810) suggested that 98.1% of the variation in shikonin yield was interpreted by the model and indicated an effective correlation between the predicted and measured values.

### 3.3. Response Surface Analysis

**Figure 3:**
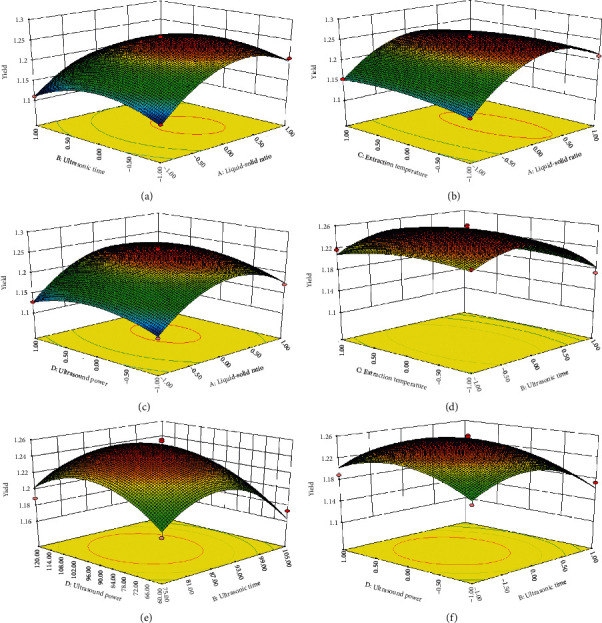
The 3D response surface of shikonin yield affected by liquid-solid ratio, ultrasonic time, extraction temperature, and ultrasonic power.

The response surface 3D map (Figure 3) showed the combined effects of different factors on the response (shikonin yield). The experimental data were fitted to equation (3), with the following optimal operating parameters: A: liquid-solid ratio, 11.27 : 1; B: ultrasonic time, 86.98 min; C: extraction temperature, 38.67°C; and D: ultrasonic power, 92.87 W.

### 3.4. Verification Tests

The optimum conditions as identified by the model were as follows: a liquid-solid ratio of 11 : 1, ultrasonic time of 87 min, extraction temperature of 39°C, and ultrasound power of 93 W. Under these conditions, the model gave predicted yield values of 1.26% ± 0.0084%. To test the validity of the RSM, the extraction was performed under the proposed conditions, and the yield was 1.25% (*n* = 5). The good correlation between these results confirmed that the response model was able to adequately reflect the expected optimization.

### 3.5. Conversion and Purification of Shikonin

The crystals obtained after alkali extraction were a dark red powder that was consistent with the color of the control product. HPLC results demonstrated the purity of the extracted samples was 96.40%.

### 3.6. Results of the MIC Determination

As shown in Table 4, shikonin reduced the growth of the bacteria. MICs for *Escherichia coli* (ATCC 25922), *S. pullorum* (C79-13), *Pseudomonas aeruginosa* (ATCC 9027), *Staphylococcus aureus* (ATCC 25923), and *Streptococcus agalactiae* (ATCC 13813) were 256, 256, 512, 128, and 128 *μ*g/mL, respectively. The MIC against isolates was higher than that against standard bacteria. The MBC for both Gram-positive and Gram-negative bacteria was equal to twofold or fourfold of MIC, which demonstrated that shikonin was a bacteriostatic agent, which was more effective against Gram-positive bacteria, with lower MIC and MBC values.

### 3.7. Time-Kill Analysis of Shikonin against Bacteria

**Figure 4:**
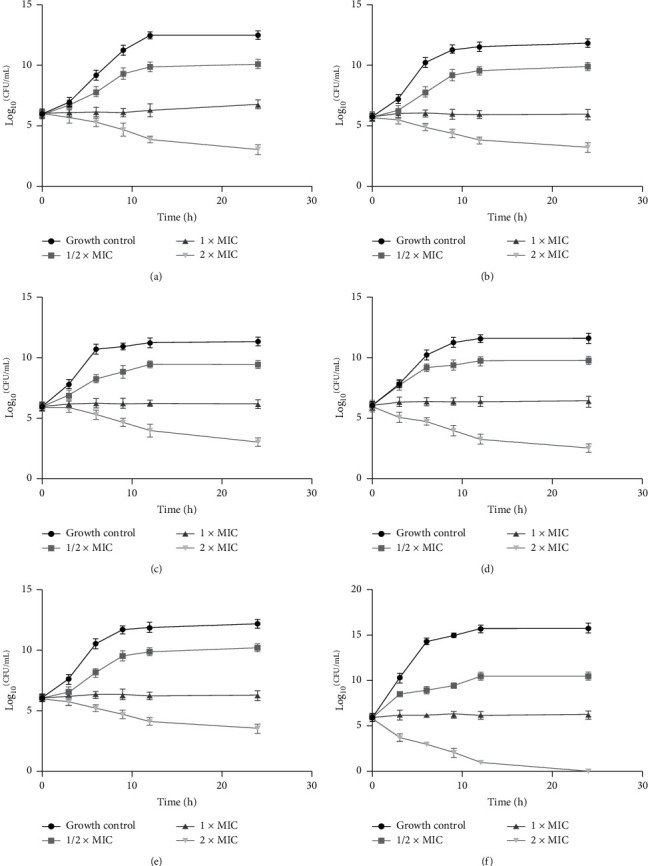
Effects of shikonin on the time-kill curve, mean ± SD: (a) *E. coli* (ATCC 25922), (b) *Salmonella pullorum* (C79-13) (c) *S. aureus* (ATCC 25923), (d) EYAC 15-12, (e) SYAC 16-10, and (f) staYAC 16-5.

Time-kill analysis of shikonin was performed on ATCC 25922, C79-13, ATCC 25923, EYAC15-12, SYAC16-10, and staYAC16-5 (as indicated in Figure 4). The number of CFU/mL over the 0 to 24 h time period against all strains at all the shikonin group was decreased more than that in the control group. After incubation for 3 h, the growth rate in the shikonin group was always lower than that of the blank control group; after treatment with the 1/2× MIC, an increasing trend was observed, but compared with that in the blank group, the growth of bacteria in the shikonin group was only mildly inhibited. All strains isolates had at least a 2-log_10_ decrease in CFU/mL with treatment with shikonin at 2× MIC at 24 h. The inhibitory effect on the standard strain of *S. aureus* (Figure 4(c)) was more obvious in the shikonin group than in the blank group. These results indicated that the antibacterial activity of shikonin showed a dose-response relationship.

## 4. Discussion

Traditional Chinese medicine extraction methods generally include infiltration methods and heat reflux methods. The extraction solvents mainly include water, ethanol, and petroleum ether [21]. The advantages of ethanol are easy removal, safe, nontoxicity, and high extraction rate; therefore, ethanol was selected as the extraction solvent for this experiment. Traditional extraction methods have certain advantages. However, these methods have disadvantages such as high cost, high risk, and serious environmental pollution, which limit their application in practice. Given the development of modern science and technology, new extraction technologies have emerged, such as UAE. Compared with the conventional extraction method, UAE greatly reduced the extraction time and improved the extraction rate. Finally, these results have provided an indication that this novel extraction method combines the advantages of UAE and RSM. This method dramatically reduced the extraction time and increased the extraction yield of the target compounds [22,23].

During the extraction process, most of the target compounds in the plant should be preserved and extracted. Many factors play important roles in the extraction process, such as the type of solvent, ratio of solvent to solid, ultrasonic extraction time, and extraction temperature [24]. Large solvent volumes can make the procedure difficult and lead to unnecessary waste, whereas small volumes may lead to incomplete extraction. From the character of total pigment, extracts obtained by microwave and hot reflux extraction were black and purple, while the control was purple, which was attributed to the high temperatures used in the system. Shikonin was easily degraded if the temperature exceeded 60°C; in view of this result, 40°C was selected as the optimal temperature for the extraction of shikonin. The results showed that the extraction yield of shikonin clearly increased with the increase in extraction time and power up to 90 min and 90 W, respectively, but these changes were not significant. This phenomenon may be explained by the ultrasonic waves induced at the beginning of ultrasonic processing, causing chaotic vibrations at the solvent-solid interface. The level of ultrasonic power controls the intensity of cavitation, which helps to release the target compounds from the plant matrix. The powerful ultrasound probably caused a large number of cavitation bubbles to form, which increased the mass transfer and interactions between the solvent and the plant matrix [25]. The ultrasonic cavitation could disrupt the cells and speed up the release and diffusion of the target compounds, thereby markedly improving the extraction yield of the target compounds to reach a maximum value. With a longer ultrasonic time and ultrasonic power, the target components were no longer released, so the extraction yield did not change noteworthily [26]. Therefore, 90 min and 90 W were selected as the ultrasonic parameters for further experiments. Dong et al. [27] purified shikonin by the alkaline hydrolysis of petroleum ether extraction solution, and the purity of shikonin obtained by this method was 75.67%. In this study, the water-soluble impurities were removed after the dissolution of petroleum ether, and high-purity shikonin (96.4%) was obtained using petroleum ether and pure water.

Shikonin has a long history of medicinal value in China; it has various pharmacological activities, including outstanding anti-inflammatory activity. *A. euchroma* has been less well studied for its antibacterial activity than its anti-inflammatory activity. In China, *A. euchroma* is mainly used as an external anti-inflammatory product such as scald ointment, which is convenient for external use and has low side effects, and has anti-inflammatory and detoxification effects. In this study, we concluded that shikonin is a bacteriostatic agent and indicated that the antibacterial effect of shikonin on *S. aureus* was more significant than that of *Escherichia coli* and *Salmonella*. Haghbeenet al. [28] concluded that shikonin was almost inactive on Gram-negative bacteria, but exerted significant bactericidal activity against Gram-positive bacteria. In general, shikonin has been shown to be active against Gram-positive bacteria, such as *S. aureus*, *Enterococcus faecium*, and *Bacillus subtilis* at MICs ranging from 0.30 to 6.25 mg/mL, as well as against various species of lactic acid bacteria. In contrast, they were inactive against Gram-negative bacteria such as *E. coli*, *Pseudomonas aeruginosa*, and *Micrococcus luteus* [29–31]. Some traditional Chinese medicines have obtained antimicrobial monomers and identified their antimicrobial action mechanisms. The main mechanisms are disrupting the integrity of bacterial cell walls and cell membranes to alter cell permeability [32,33], altering the synthesis of cellular proteins and nucleic acids [34], and inhibiting the activity of bacterial enzymes [35]. It was speculated that the disruption of the cell wall integrity of *S. aureus*, in conjunction with the following experiments, could determine the antibacterial mechanism of shikonin against *S. aureus.* In this study, the results of the antibacterial experiments showed that shikonin had poor solubility and was less sensitive to bacteria; therefore, these results provided a reference for the development of shikonin-related preparations and the prevention and treatment of bacterial infections caused by *S. aureus*. The experiments showed that these results can prove that shikonin has antibacterial activity and provide a basis for the study of the antibacterial activity of shikonin.

## 5. Conclusions

In this study, a green, economic, and efficient ultrasonic extraction method was established to extract shikonin from *A. euchroma*. Further, the results of the antibacterial test suggested that shikonin exerted significant antibacterial activity. In addition, shikonin showed greater inhibitory potential against Gram-positive bacteria. This study confirmed that shikonin has a certain degree of antibacterial activity and inhibits the growth of bacteria in a dose-dependent manner.

## Figures and Tables

**Table 1:** Independent factors and their levels used in ultrasonic extraction.

Independent factors	Symbol	Levels		
		−1	0	1
Liquid-solid ratio	A	5 : 1	10 : 1	15 : 1
Ultrasonic time (min)	B	75	90	105
Extraction temperature (°C)	C	30	40	50
Ultrasonic power (W)	D	60	90	120

**Table 2:** Box–Behnken design for independent variables and observed responses.

Run no.	Experimental design				Dependent variables
	Liquid-solid ratio	Ultrasonic time	Extraction temperature	Ultrasonic power	Yield (%)
1	−1	−1	0	0	1.122
2	1	−1	0	0	1.206
3	−1	1	0	0	1.11
4	1	1	0	0	1.157
5	0	0	−1	−1	1.224
6	0	0	1	−1	1.191
7	0	0	−1	1	1.213
8	0	0	1	1	1.229
9	−1	0	0	−1	1.12
10	1	0	0	−1	1.172
11	−1	0	0	1	1.128
12	1	0	0	1	1.198
13	0	−1	−1	0	1.221
14	0	1	−1	0	1.172
15	0	−1	1	0	1.217
16	0	1	1	0	1.18
17	−1	0	−1	0	1.137
18	1	0	−1	0	1.211
19	−1	0	1	0	1.154
20	1	0	1	0	1.19
21	0	−1	0	−1	1.18
22	0	1	0	−1	1.173
23	0	−1	0	1	1.189
24	0	1	0	1	1.175
25	0	0	0	0	1.253
26	0	0	0	0	1.26
27	0	0	0	0	1.247
28	0	0	0	0	1.25
29	0	0	0	0	1.258

**Table 3:** ANOVA of response surface quadratic model analysis for the extraction yield.

Source	Sum of squares	Degrees of freedom	Mean square	*F*-value	*p* value prob > *F*
Model	0.051	14	3.67*E* − 03	51.64	<0.0001^a^
*A*	0.011	1	0.011	154.72	<0.0001
*B*	2.35*E* − 03	1	2.35*E* − 03	33.14	<0.0001
*C*	2.41*E* − 05	1	2.41*E* − 05	0.34	0.5695
*D*	4.32*E* − 04	1	4.32*E* − 04	6.09	0.0271
*AB*	3.42*E* − 04	1	3.42*E* − 04	4.82	0.0454
*AC*	3.61*E* − 04	1	3.61*E* − 04	5.09	0.0406
*AD*	8.10*E* − 05	1	8.10*E* − 05	1.14	0.3035
*BC*	3.60*E* − 05	1	3.60*E* − 05	0.51	0.488
*BD*	1.23*E* − 05	1	1.23*E* − 05	0.17	0.6841
*CD*	6.00*E* − 04	1	6.00*E* − 04	8.46	0.0115
*A* ^2^	0.029	1	0.029	404.78	<0.0001
*B* ^2^	0.011	1	0.011	160.64	<0.0001
*C* ^2^	9.81*E* − 04	1	9.81*E* − 04	13.83	0.0023
*D* ^2^	6.10*E* − 03	1	6.10*E* − 03	86	<0.0001
Residual	9.94*E* − 04	14	7.10*E* − 05		
Lack of fit	8.76*E* − 04	10	8.76*E* − 05	2.99	0.1512^b^

^a^Significant. ^b^Not significant.

**Table 4:** Antibacterial activities of shikonin against G^+^ and G^−^ bacteria.

Strains	MIC (*μ*g/mL)	MBC (*μ*g/mL)
G^+^		
*E. coli* (ATCC 25922)	256	512
*Salmonella* (C79-13)	256	1024
*P. Aeruginosa* (ATCC 9027)	512	1024
*E. coli* isolates	512∼1024	1024∼2048
*Salmonella* isolates	128∼512	256∼1024

G^−^		
*S. agalactiae* (ATCC 13813)	128	256
*S. Aureus* (ATCC 25923)	128	256
*S. aureus* isolates	512∼1024	1024∼2048

## Data Availability

The data such as single-factor experiment, MICs, and MBCs to support the findings of this study are included within the article. Other data used to support the findings of this study are available from the corresponding author upon request.

## References

[1] Andújar I., Ríos J., Giner R., Recio M. (2013). Pharmacological properties of shikonin–a review of literature since 2002. *Planta Medica*.

[2] Chen X., Yang L., Oppenheim J. J., Howard O. M. Z. (2002). Cellular pharmacology studies of shikonin derivatives. *Phytotherapy Research*.

[3] shi-ying Z., Rong W., Yan-qing L., Cai-ming Y., Da-you F. (2011). Ultrasonic wave-microwave collaborative extraction of shikonin and its derivatives and preparation of shikonin. *China Food Additives*.

[4] Hua-geng Z., Xiao-hua L., Zhi-jun F. (2015). Studies of the optimum extraction process of AIkannin from Arnebia Eu-chroma (RoyeI) Johnst in Xinjiang. *Strait Pharmaceutical Journal*.

[5] Lu-Rong Y., Xian-zhang H. (2011). Extraction technology of *Arnebia euchroma* (*Royle*) *Johnst*. *Chinese Journal of Spectroscopy Laboratory*.

[6] Xie X., Qiu M. (1997). Study on the extracting procedures of radix *Arnebiae Seu Lithospermi* in different preparations. *China Pharmacy*.

[7] Liu T. T., Ma C.-h., Sui X.-y. (2012). Preparation of shikonin by hydrolyzing ester derivatives using basic anion ion exchange resin as solid catalyst. *Industrial Crops & Products*.

[8] Cur X. (2014). Extraction of shikonin with surfactant-assisted ultrasonic from Arnebia euchroma. *Asian Journal of Chemistry: An International Quarterly Research Journal of Chemistry*.

[9] Wang L., Li T., Liu F. (2019). Ultrasonic-assisted enzymatic extraction and characterization of polysaccharides from dandelion (*Taraxacum officinale*) leaves. *International Journal of Biological Macromolecules*.

[10] Fogaça F. H. S., Trinca L. A., Bombo Á. J., Sant’Ana L. S. (2013). Optimization of the surimi production from mechanically recovered fish meat (MRFM) using response surface methodology. *Journal of Food Quality*.

[11] Bezerra M. A., Santelli R. E., Oliveira E. P., Villar L. S., Escaleira L. A. (2008). Response surface methodology (RSM) as a tool for optimization in analytical chemistry. *Talanta*.

[12] Sodeifian G., Saadati Ardestani N., Sajadian S. A. (2019). Extraction of seed oil from diospyros lotus optimized using response surface methodology. *Journal of Forestry Research*.

[13] Yang J.-X., Hong G.-B. (2019). Optimized extraction for active compounds in Glossogyne tenuifolia using response surface methodology. *Journal of Food Measurement and Characterization*.

[14] Parray J. A., Hamid R., Kamili A. N., Shameem N., Jan S., Ganai B. A. (2015). Biological efficacy and radical scavenging potential of shikonin in *Arnebia benthamii* (Wall ex. G Don) Johnston. *Industrial Crops and Products*.

[15] Zhou J. (2016). *Study on the Extraction, Conversion and Liposome Preparation of Shikonin*.

[16] Zhang Y., Wu Y.-T., Zheng W. (2017). The antibacterial activity and antibacterial mechanism of a polysaccharide from *Cordyceps cicadae*. *Journal of Functional Foods*.

[17] Seanego C. T., Ndip R. N. (2012). Identification and antibacterial evaluation of bioactive compounds from *Garcinia kola* (Heckel) seeds. *Molecules*.

[18] Carson C. F., Mee B. J., Riley T. V. (2002). Mechanism of action of *Melaleuca alternifolia* (tea tree) oil on *Staphylococcus aureus* determined by time-kill, lysis, leakage, and salt tolerance assays and electron microscopy. *Antimicrob Agents Chemother*.

[19] Keepers T. R., Gomez M., Celeri C., Nichols W. W., Krause K. M. (2014). Bactericidal activity, absence of serum effect, and time-kill kinetics of ceftazidime-avibactam against beta-lactamase-producing enterobacteriaceae and *Pseudomonas aeruginosa*. *Antimicrobial Agents and Chemotherapy*.

[20] Chen Y., Yin L. Z., Zhao L. (2017). Optimization of the ultrasound-assisted extraction of antioxidant phloridzin from *Lithocarpus polystachyus* Rehd. using response surface methodology. *Journal of Separation Science*.

[21] Ai-ping W., Mao-dong Z., Zhen L. (2013). Research survey of the shikonin the extraction process and the relevant preparations. *Guide of China Medicine*.

[22] Li C., Zhang Y., Zhao C. (2017). Ultrasonic assisted-reflux synergistic extraction of camptothecin and betulinic acid from camptotheca acuminata decne. fruits. *Molecules*.

[23] Wang X., Jiang Y., Hu D. (2016). Optimization and in vitro antiproliferation of curcuma wenyujin’s active extracts by ultrasonication and response surface methodology. *Chemistry Central Journal*.

[24] Jiang Y., Li P., Li S. P., Wang Y. T., Tu P. F. (2007). Optimization of pressurized liquid extraction of five major flavanoids from *Lysimachia clethroide*. *Journal of Pharmaceutical and Biomedical Analysis*.

[25] Mason T., Vinatoru M. (2011). The extraction of natural products using ultrasound or microwaves. *Current Organic Chemistry*.

[26] Sahin S., Samli R. (2013). Optimization of olive leaf extract obtained by ultrasound-assisted extraction with response surface methodology. *Ultrasonics Sonochemistry*.

[27] Dong H. R. (2004). *Study on the Technological Processes in Shikonin of Extracting, Convertion, and Purification*.

[28] Haghbeen K., Pourmolaei S., Mareftjo M. J. (2011). Detailed investigations on the solid cell culture and antimicrobial activities of the Iranian Arnebia euchroma. *Journal of Biomedicine and Biotechnology*.

[29] Papageorgiou V. P., Assimopoulou A. N., Couladouros E. A., Hepworth D., Nicolaou K. C. (1999). The chemistry and biology of alkannin, shikonin, and related naphthazarin natural products. *Angewandte Chemie International Edition*.

[30] Shen C. C., Syu W. J., Li S. Y., Lin C. H., Lee G. H., Sun C. M. (2002). Antimicrobial activities of naphthazarins from *Arnebia euchroma*. *Journal of Natural Products*.

[31] Li H. M., Tang Y. L., Zhang Z. H. (2012). Compounds from *Arnebia euchroma* and their related anti-HCV and antibacterial activities. *Planta Medica*.

[32] Plaper A., Golob M., Hafner I., Oblak M., olmajer T., Jerala R. (2003). Characterization of quercetin binding site on DNA gyrase. *Biochemical & Biophysical Research Communications*.

[33] Wu D., Kong Y., Han C. (2008). d-Alanine:d-alanine ligase as a new target for the flavonoids quercetin and apigenin. *International Journal of Antimicrobial Agents*.

[34] Wang H. T. (2009). *Anti-microoganism Activity and Anti-microbial Mechanism of Soybean Isoflavone*.

[35] Sun X., Wang J. C., Li H. T., Du G. C., Guo D. S. (2005). A study on the antibacterial mechanism of rosmarinic acid. *Journal of Qingdao University (Natural Science Edition)*.

